# Characteristics and Treatment Challenges of Non-Clear Cell Renal Cell Carcinoma

**DOI:** 10.3390/cancers13153807

**Published:** 2021-07-28

**Authors:** Pierangela Sepe, Arianna Ottini, Chiara Carlotta Pircher, Andrea Franza, Melanie Claps, Valentina Guadalupi, Elena Verzoni, Giuseppe Procopio

**Affiliations:** Department of Medical Oncology Unit, Fondazione IRCCS Istituto Nazionale dei Tumori di Milano, 20133 Milan, Italy; arianna.ottini@istitutotumori.mi.it (A.O.); chiara.pircher@istitutotumori.mi.it (C.C.P.); andrea.franza@istitutotumori.mi.it (A.F.); melanie.claps@istitutotumori.mi.it (M.C.); valentina.guadalupi@istitutotumori.mi.it (V.G.); elena.verzoni@istitutotumori.mi.it (E.V.); giuseppe.procopio@istitutotumori.mi.it (G.P.)

**Keywords:** renal cancer, non-clear cell RCC, targeted therapy, immunotherapy

## Abstract

**Simple Summary:**

Non-clear cell renal cell carcinomas (ncRCC) make up a heterogeneous group subclassified into different subtypes that differ in genetic and biochemical characteristics from each other and from ccRCC. ncRCC are a rare finding in clinical practice, and no standard-of-care has yet been established. Treatment choices are in fact based on extrapolating results from clear cell RCC trials, retrospective data, or case reports. The aim of this review is to supplyt precise recommendations for each histological type focusing on pathogenetic mechanisms of nc-RCC, summarizing the therapeutic strategies adopted over the last few decades, and exploring the emerging role of immunotherapy and new targeted drugs as future potential treatment options.

**Abstract:**

Non-clear cell renal cell carcinomas (RCC) comprise several rare and poorly described diseases, often characterized by bad prognosis and with no standard treatments available. The gap in their clinical management is linked to the poor molecular characterization in handling the treatment of non clear-cell RCC with untailored therapies. Due to their rarity, non-clear RCC are in fact under-represented in prospective randomized trials. Thus, treatment choices are based on extrapolating results from clear cell RCC trials, retrospective data, or case reports. Over the last two decades, various options have been considered as the mainstay for the treatment of metastatic RCC (mRCC), including angiogenesis inhibitors, vascular endothelial growth factor receptor inhibitors, other tyrosine kinase inhibitors (TKIs), as well as MET inhibitors and mammalian targeting of rapamycin (mTOR) inhibitors. More recently, the therapeutic armamentarium has been enriched with immunotherapy, alone or in combination with targeted agents that have been shown to significantly improve outcomes of mRCC patients, if compared to TKI single-agent. It has been widely proven that non-clear cell RCC is a morphologically and clinically distinct entity from its clear cell counterpart but more knowledge about its biology is certainly needed. Histology-specific collaborative trials are in fact now emerging to investigate different treatments for non-clear cell RCC. This review summarizes pathogenetic mechanisms of non-clear cell RCC, the evolution of treatment paradigms over the last few decades, with a focus on immunotherapy-based trials, and future potential treatment options.

## 1. Introduction

Renal cell carcinoma (RCC) accounts for 3% to 4% of all adult malignancies [1]. Among all the histologic variants, clear cell RCC (ccRCC) is the most common, representing from 70% to 90% of all renal carcinomas. The remaining cases are categorized as non-clear cell carcinomas (ncRCC). This heterogeneous group is further subclassified into different subtypes, including papillary (10–15% of all RCCs), chromophobe (5% to 7%), collecting duct (1% to 2%), renal medullary (<1%), and translocation RCC tumors (<1%) [2]. The fourth edition of the World Health Organization (WHO) classification of urogenital tumors (WHO “blue book”), published in 2016, recognized new epithelial renal tumors with low incidence rates such as hereditary leiomyomatosis and renal cell carcinoma (RCC) syndrome-associated RCC, succinate dehydrogenase-deficient RCC, tubulocystic RCC, acquired cystic disease-associated RCC, and clear cell papillary RCC [3]. All these histologies differ for genetic and biochemical characteristics from each other and from ccRCC [4].

Due to its rarity and poor molecular characterization, ncRCC are often managed with untailored treatments. These tumors are in fact under-represented in prospective randomized trials. Thus, treatment choices are based on extrapolating results from ccRCC trials, retrospective data, or case reports. Over the last two decades, various options have been considered as the mainstay for the treatment of metastatic RCC (mRCC), including angiogenesis inhibitors, vascular endothelial growth factor receptor inhibitors, other tyrosine kinase inhibitors (TKIs), as well as mesenchymal–epithelial transition factor gene (MET)-inhibitors and mammalian target of rapamycin (mTOR) inhibitors. More recently, the therapeutic armamentarium has been enriched with immunotherapy, alone or in combination with targeted agents that were shown to significantly improve outcomes of mRCC patients compared to TKI single-agent [5,6,7,8,9,10]. More knowledge about the biology of non-clear histologies is certainly needed, but it is now widely proven that ncRCC is a morphologically and clinically distinct entity from its clear cell counterpart. Histology-specific collaborative trials and controlled biomarker-based clinical trials are in fact now underway to investigate different treatments for ncRCC. This review summarizes pathogenetic mechanisms of ncRCC, the evolution of treatment paradigms over the last few decades, and future potential treatment options.

## 2. Pathogenetic Mechanisms

### 2.1. Papillary

Papillary renal cell carcinoma (PRCC) is the most common type of ncRCC (10–15% of all RCCs. PRCC originates from proximal and distal convoluted tubules, contrary to clear cell RCC, which may arise only from proximal tubules of the kidney [11]. The diagnosis is usually incidental and the clinical behavior is less aggressive than the clear cell counterpart. Among RCC subtypes, papillary RCC is most frequently found to be multifocal and bilateral [11]. Histologically, PRCC demonstrates discrete papillary architecture with fibrovascular cores lined by neoplastic cells. PRCC is usually divided into histological (type 1 or 2) or molecular (MET driven or MET independent) subgroups [12]. Type 1 papillary RCC has better clinical outcome than type 2 papillary RCC [12]. In the last few years, there has been remarkable progress in the comprehension of the molecular basis of PRCC, aiming to better understand the heterogeneity of this disease and the distinct response to the provided treatments. Different studies have revealed that type 2 PRCCs represent a diversified group of ncRCC which may be subdivided into additional subtypes based on the genetic and molecular characteristics of these tumors, reflecting a different clinical course and outcome [12,13,14,15]. Type 1 tumors were associated with *MET* alterations, whereas type 2 tumors were characterized by *CDKN2A* silencing, *SETD2* mutations, *TFE3* fusions, and increased expression of the *NRF2*-antioxidant response element (ARE) pathway [13]. The role of CDKN2A alterations as an independent prognostic marker associated with type 2 tumors requires validation. On the other hand, this study suggests that gene fusions involving TFE3 or TFEB are underappreciated in type 2 tumors and should be considered. A CpG island methylator phenotype (CIMP) was observed in a distinct subgroup of type 2 papillary renal-cell carcinoma and resulted in associated poor survival and mutation of the gene encoding fumarate hydratase (FH), which is an enzyme of the Krebs cycle acting as a catalyst for the conversion from fumarate to malate [13]. Papillary RCCs are distinguished from their clear cell counterpart as they show different cytogenetic alterations; in particular Kovacs et al. found that PRCC is characterized by trisomy of chromosomes 3q, 7, 8, 12, 16, 17, or 20, and in men by loss of the Y chromosome [16]. Papillary type 2 tumors appear to frequently gain 8q and lose 1p and 9p [17].

Much of our knowledge of the genetic basis of PRCC lies in the study of the inherited form of the disease, described for the condition known as hereditary papillary renal cell cancer. Hereditary PRCC is a rare familial disorder associated with an increased risk of multiple and bilateral renal tumors, mostly harboring morphological characteristics typical of type 1 papillary cancer [18]. Germline *MET* proto-oncogene alterations are the hallmark of this syndrome, and these are rarely observed in sporadic forms [19].

Previously published next-generation sequencing studies have identified several mutated genes associated with PRCC. Recently, a report from The Cancer Genome Atlas Research Network described the results of a comprehensive molecular profiling of 161 primary papillary renal-cell carcinomas, using whole-exome sequencing, copy-number analysis, messenger RNA and microRNA sequencing, DNA-methylation analysis, and proteomic analysis [13]. Type 1 and type 2 papillary renal-cell carcinomas were proven to be different types of renal cancer characterized by specific genetic alterations [13].

*MET* is deregulated in many types of human malignancies, including cancers of kidney, liver, stomach, breast, and brain [20]. The *MET* pathway, aberrantly activated particularly in type 1 PRCC, is associated with tumor growth, angiogenesis, and promotion of metastases, as well as treatment resistance. Several additional pathways including RAS, PI3K, Stat, beta-catenin and Notch pathways may also be activated [21,22]. In MET-drive cases of PRCC, MET inhibition may be a targeted treatment approach and targeted therapies have been tested in different clinical trials [22,23,24,25,26,27,28]. Given the limited response of PRCC to the conventional treatment strategy used in ccRCC, MET-targeted therapy alone or combination with other agents could provide better outcomes for these patients. Genetic and cytogenetic alterations in ncRCC aressumarized in Table 1.

### 2.2. Chromophobe

Chromphobe RCC (chRCC) is a rare type of kidney cancer accounting for 5% to 7% of all RCCs. This tumor originates more distally within the nephron, if compared to other kidney cancers with more proximal origins. chRCC are low malignant tumors with a 5–6% risk of metastasis. Most tumors are sporadic but a part may be associated with bilateral multifocal Birt–Hogg–Dubb Syndrome, an autosomal dominant genodermatosis characterized by the development of small dome-shaped papules on the face, neck, and upper trunk (fibrofolliculomas) [29]. In addition, Cowden syndrome with germline mutations in PTEN is associated with a higher incidence of chromophobe-like or oncocytoma-like neoplasms. A study was conducted to elucidate the genetic lesions of eosinophilic chromophobe renal cell carcinoma comparing them with those found in classic chromophobe renal cell carcinoma and in renal oncocytoma. Classic chromophobe renal cell carcinomas showed losses of multiple chromosomes from among chromosomes 1, 2, 6, 10, and 17, and this pattern of genetic abnormality is not present in renal oncocytoma [30]. Comprehensive genomic analyses of chRCC demonstrated a low somatic mutation identifying *TP53* and *PTEN* as the most frequently mutated genes [31]. Mutation on the short arm of chromosome 7 in chromophobe was shown to lead to a loss of folliculin tumor suppressor gene with mTOR and c-kit activation [31]. Genetic and cytogenetic alterations in ncRCC aressumarized in Table 1.

### 2.3. Collecting Duct Carcinoma

Collecting duct carcinoma (CDC) originates from the epithelium of the distal collecting ducts of the kidney. The diagnosis is usually incidental and in most cases at advanced stages. Prognosis is very poor, with a 2-year overall survival rate of approximately two-thirds of cases. Lymph nodes, lungs, liver, bones, and adrenal glands are frequent sites of metastases. The histopathologic and immunohistochemical analyses are fundamental to differ CDC from urothelial carcinoma and renal cell carcinoma. CDC present a tubulopapillary architecture, a marked stromal dysplasia, and a high nuclear grade; immunohistochemistry reveals the presence of high-molecular weight cytokeratins and Ulex europaeus agglutinin 1 lectin, frequently co-expressed with vimentin [32]. Pathognomonic molecular features of CDC are still lacking, as only a few studies have explored the molecular profile of CDC. Here, we report the most significant case studies.

Pal et al. performed a comprehensive genomic profiling of 17 CDC cases identifying clinically relevant somatic mutations in *NF2*, *SETD2,* and *SMARCB1* genes with 29%, 24%, and 18% frequency, respectively. In particular, mTOR inhibitors may be of interest in patients with NF2 alterations [33]. Another genomic profiling performed on seven CDC cases reported homozygous deletion of *CDKN2A* gene and a recurrent mutation in the *MLL* gene in about 50% of cases. Moreover, a dysregulation of several solute carrier family genes including tumor over-expression of *SLC7A11*, a cisplatin-resistance marker, was observed [34]. Another RNA-sequencing study on 11 CDC and nine upper-tract urothelial carcinoma samples compared with ccRCC histology showed that CDC tumors clustered separately from other tumor types. Furthermore, this study defined CDC as a metabolic disease characterized by a shift toward aerobic glycolysis and an over-expression of immune genes related to lymphocyte activation and T cell proliferation [35]. An Italian work recently published, showed that CDC is a molecularly heterogeneous disease composed of at least two subtypes distinguished by cell signaling and metabolic and immune-related alterations. The identification of these distinct subtypes and their transcriptomic traits provides the rationale for patient stratification and alternative therapeutic approaches. These insights could lead to rationalize the use of targeted therapies or immunotherapy for rare tumors according to the individual genomic alterations harbored [36]. Genetic and cytogenetic alterations in ncRCC aressumarized in Table 1.

### 2.4. Sarcomatoid

Sarcomatoid RCC are not defined as a distinct entity. A sarcomatoid dedifferentiation can be present in up to 20% of mRCC patients. The presence of this histologic feature is associated with high grade, aggressive tumors and short survival [37,38]. Sarcomatoid tumors are very inflamed tumors presenting high expression of PD-L1 [37,38]. Genetic and cytogenetic alterations in ncRCC aressumarized in Table 1.

### 2.5. Other Histological Types

The 2016 WHO classification of urogenital tumors (WHO “blue book”) include other renal tumors with low incidence rates. Among them, we mention Xp11 translocation RCC, Hereditary leiomyomatosis, renal cell carcinoma, and renal medullary carcinoma.

Xp11 translocation RCC have been recently recognized as a subset of RCC. These tumors are characterized by chromosome translocations involving the Xp11.2 breakpoint and resulting in gene fusions involving the TFE3 transcription factor gene that maps to this locus. This a subtype affects at least one-third of pediatric RCCs and for 15% of RCCs in patients <45 years of age. Clinical behavior is aggressive with widespread systemic metastases. Prognosis is poor. Immunohistochemistry using antibodies against TFE3 (C-terminal part of transcription factor binding to IGHM enhancer 3) confirmed the diagnosis of Xp11 translocation RCC [39].

Hereditary leiomyomatosis and renal cell carcinoma, also known as HLRCC, is a rare genetic disorder characterized by smooth muscle growths (leiomyomas) on the skin and uterus and an increased risk of developing kidney cancer [40]. Activity of fumarate hydratase is reduced or absent in tumors developing in individuals with leiomyomatosis [40]. The consequent fumarate-accumulation in these tumors generates a pseudo-hypoxic state with Hypoxia-Inducible Factor (HIF)-1 up-regulation. HIF-1 is a transcription factor involved in homeostasis, vascularization, anaerobic metabolism, and immunological responses [40].

Renal medullary carcinoma (RMC) is another subset of RCC characterized by aggressive clinical behavior and poor prognosis. This tumor typically affects young adults and is almost exclusively associated with sickle cell trait. RMC tumors usually express cytokeratin AE1/AE3, low molecular weight cytokeratin, vimentin, hypoxia-inducible factors (HIF), and vascular endothelial growth factor (VEGF). Mariño-Enríquez et al. reported anaplastic lymphoma kinase (ALK) receptor tyrosine kinase rearrangement in RMC suggesting a rationale for studying the treatment of RMC with targeted ALK inhibitors [41]. Genetic and cytogenetic alterations in ncRCC aressumarized in Table 1.

## 3. Therapies

### 3.1. Papillary

To date, we have no approved therapy specifically indicated for papillary RCC (PRCC). Data concerning ncRCC treatment is derived from studies on ccRCC, retrospective series, expanded access programs or case reports [42,43,44]. Angiogenesis represents a key pathogenic mechanism of mRCC. However, most of the prospective trials investigating antiangiogenic treatments as VEGF inhibitors included predominantly patients with clear cell component.

A post hoc analysis of the global ARCC trial reported that 30 and 25 patients with metastatic PRCC (mPRCC) were treated with interferon-alfa (IFN-α) and temsirolimus, respectively with a hazard ratio (HR) for death of 0.50 (95% CI = 0.27, 0.94) [45]. Since this result, everolimus was used as best comparator for subsequent studies. The ESPN trial compared everolimus to sunitinib in all subtypes of ncRCC (27 papillary, 12 chRCC, 10 unclassified, 7 translocation, 12 sarcomatoid) [46]. A cross-over between the two arms upon disease progression was allowed [46]. The results were not so encouraging with only three partial responses registered administering sunitinib in the first line setting and one with everolimus [46]. The ASPEN trial is another study comparing everolimus to sunitinib in all subtypes of ncRCC [47]. This study included 70 papillary of which 6 had papillary type 1, 16 chRCC, 8 translocation, 22 unclassified, and 16 sarcomatoid RCC [47]. No statistically significant differences in overall-survival (OS) and progression-free survival (PFS) were observed. Exploratory analysis of this study showed that patients with good risk disease had a better PFS when treated with sunitinib [47]. Moreover, different responses for subtypes were observed with a more favorable PFS in patients with papillary and unclassified histologies receiving sunitinib [47]. On the other hand, everolimus showed a better PFS in patients with chRCC subtypes [47].

Several retrospective studies reported partial responses with sunitinib in mPRCC. A retrospective analysis conducted by Choueri et al. included 41 mPRCC patients and 12 metastatic patients with chRCC histology who received sunitinib or sorafenib as their initial TKI treatment. PFS for the papillary cohort was 8.6 months. The two papillary patients achieving partial response were treated with sunitinib, and no responders were observed in the sorafenib group. Sunitinib-treated papillary patients had a PFS of 11.9 months compared with 5.1 months for sorafenib-treated patients (*p* < 0.001) [43]. In a phase II study of sunitinib conducted by Tannir et al. no patients with mPRCC (of 27 included) had a partial response, with a PFS of less than 3 months [48]. The phase II prospective trial SUPAP gave more encouraging results, showing activity of sunitinib in both type 1 and 2 mPRCC. In detail, 15 and 46 patients respectively with type 1 and type 2 mPRCC were enrolled. Both PFS and OS were longer in type 1 PRCC. Two patients with type 1 and five with type 2 disease experienced a partial response with a median PFS of 6.6 months and 5.5 months in type 1 and type 2 respectively [49]. *MET* expression level was high across all PRCC. 

As described above, activating mutations or amplifications in *MET* are common in patients with PRCC [20]. For this reason, *MET* inhibitors have been tested in different clinical trials with promising findings. The objective response rate (ORR) observed in *MET*-driven mPRCC was variable between 18% and 50%, whereas no meaningful activity was observed in *MET*-independent tumors. Foretinib, an oral inhibitor targeting *MET*, *VEGF*, *RON*, *AXL,* and *TIE-2* receptors, demonstrated activity, a manageable toxicity profile and a high response rate in mPRCC patients with germline *MET* mutations [22]. The presence of a germline *MET* mutation was highly predictive of a response. All 10 patients included with germline mutation experienced a partial response (*n* = 5) or stable disease (*n* = 5). In contrast, only one out of five patients with somatic *MET* mutation had a partial response, while no responses were seen in patients with *MET* amplification (*n* = 2), and only one out of 18 patients with a gain of chromosome 7 experienced a partial response [22]. Crizotinib, a *MET*, *ROS1,* and *ALK* inhibitor, was shown to be active and well tolerated in advanced PRCC type 1, achieving ORR and long-lasting disease control in patients with *MET* mutations or amplification [23]. In a single-arm multicenter phase II study 109 patients with mPRCC were treated with savolitinib, a highly selective *MET* TKI. Overall, 40% of treated patients had *MET*-driven tumor, as defined by chromosome 7 copy gain, focal *MET* or *HGF* gene amplification, or *MET* kinase domain mutations. Median PFS was 6.2 months and 1.4 months in patients with *MET* dependent and *MET* independent tumors, respectively [24]. Moreover, savolitinib was tested in the phase III trial SAVOIR, which unfortunately was prematurely closed in 2019 due to discouraging results. This randomized trial was designed to assess the efficacy and safety of savolitinib versus sunitinib in patients with *MET*-driven, unresectable, locally advanced or mPRCC. The trial did not meet its primary endpoint of improving PFS: median PFS was not statistically different between the two groups (7.0 months vs. 5.6 months HR 0.71). However, savolitinib was better tolerated, with fewer grade 3–4 adverse events registered. Moreover, savolitinib-responding patients showed a longer response than sunitinib, providing valuable data to conduct biomarker driven therapy in this specific challenging population [25]. Based on recent evidence, cabozantinib, an oral TKI with activity against a broad range of targets, including *MET*, *RET*, *AXL*, *VEGFR2*, *FLT3*, and *c-KIT*, could be recommended for the treatment of mPRCC. A retrospective study, including 112 ncRCC patients, of whom 60% with papillary histology, supports the antitumor activity and safety of cabozantinib across ncRCC with ORR of 27% across the entire cohort [26]. A retrospective analysis conducted by Campell et al. on 30 patients with ncRCC, 57% of whom had a PRCC, showed ORR of 14.3% [28]. The PAPMET study was the first randomized trial specific to mPRCC [27]. This randomized multicentric phase II trial tested four different *MET* inhibitors in mPRCC: cabozantinib, crizotinib, savolitinib, and sunitinib [27]. However, due to futility analysis, the protocol was revised and the crizotinib and savolitinib arms were prematurely closed. Recently published results showed a significantly longer PFS with cabozantinib treatment (median 9.0 months, 95% CI 6–12) compared with sunitinib group (5.6 months, 95% CI 3–7; hazard ratio for progression or death 0.60, CI 0.37–0.97, one-sided *p* = 0.019) [27]. Further studies exploring the combination of *MET* inhibitors with immunotherapeutic agents are ongoing.

RCC treatment paradigms have dramatically changed in the last few years since immunotherapy approval. Retrospective trials evaluated the role of checkpoint inhibitors in ncRCC and several trials are ongoing in this setting as well. Retrospective series on ncRCC including papillary, chRCC, unclassified, CDC, Xp11 translocation, and ccRCC with sarcomatoid differentiation treated with anti PD-1 or PDL-1, alone or in combination with cytotoxic T-lymphocyte associated protein-4 (CTLA-4), reported ORR of 19–20% [50,51]. Responses were higher for patients presenting with sarcomatoid or rhabdoid differentiation [50,51]. A retrospective study including 18 ncRCC patients treated with the combination nivolumab–ipilimumab reported ORR of 28% [52]. Among the prospective evidence, KEYNOTE-427 was a phase II single arm trial evaluating pembrolizumab in the first line setting of RCC. This trial provided a separate cohort for ncRCC (cohort-B) including 165 patients (71.5% papillary, 12.7% chRCC, 15.8% unclassified). Among patients with papillary, chRCC, and unclassified histology, reported ORR were 28%, 9.5%, and 30.8%. Among 38 patients with sarcomatoid differentiation ORR was higher (42.1%) [53].

The CALYPSO trial enrolled 42 patients with mPRCC. All patients received savolitinib in combination with durvalumab (PD-L1 inhibitor) with ORR of 29% [54].

Completed and ongoing clinical trials evaluating treatments for mPRCC patients are summarized in Table 2 and Table 3.

### 3.2. ChRCC

To date, we have no approved therapy specifically indicated for chRCC. Due to its rarity, data concerning the treatment of chRCC histology is derived from studies including other ncRCC subtypes. As described above, patients with chRCC RCC may benefit from mTHOR inhibitors. The reason is linked to a mutation on chromosome 7 commonly described in chRCC tumors that was shown to lead to a loss of folliculin gene with upregulation of mTOR. The ASPEN trial included 16 patients with chRCC. Everolimus was shown to be superior to sunitinib in terms of ORR (33.3 vs. 10%) and PFS (11.4 vs. 5.5 months). On the other hand, OS was longer in the sunitinib arm compared to everolimus (31.5 vs. 13.2 months) [47]. Moreover, 12 patients with chRCC were randomized in the ESPN trial. This trial showed a benefit in OS toward sunitinib with 31.9 vs. 25.1 months for everolimus [46]. In the retrospective analysis conducted by Choueri et al., sorafenib was shown to be superior over sunitinib in terms of PFS and ORR in 12 patients with chRCC histology [43]. The phase II study conducted by Tannir et al. was shown in the five patients with chRCC histology, median PFS of 12.7 months and ORR of 40% with only two partial responses [48]. A prospective study conducted by Procopio et al. evaluating efficacy of sorafenib in ncRCC included three patients with chRCC. No data were available regarding OS and PFS. No partial or complete responses were observed [55]. Cabozantinib showed ORR of 16.6% in six patients with ChRCC histology included in a retrospective analysis conducted by Campbell et al. [28] Moreover, the retrospective analysis exploring the efficacy of PD-1 or PDL-1, alone or in combination with anti CTLA-4 in ncRCC, showed no objective response in the 10 patients with chRCC. Data were not available regarding PFS whereas OS was not reached [50]. The retrospective series evaluating activity of nivolumab in ncRCC included five patients with chRCC. No responses were observed [51]. A number of 21 patients with chRCC were treated with pembrolizumab monotherapy in the KEYNOTE-427. OS was not reached; median PFS was 4.1 months for the whole group. As reported above, ORR for chRCC was 9.5% [53].

### 3.3. CDC

To date, we have no approved therapy specifically indicated for metastatic CDC (mCDC). Evidence from randomized histology-specific clinical trials evaluating the best treatment for patients with mCDC are lacking. One of the major findings in terms of overall response rate was achieved in a prospective phase II trial showing the efficacy of the chemotherapy combination platinum-based plus gemcitabine in 23 previously untreated mCDC patients [56]. Here, 23 patients with mCDC were treated with gemcitabine and cisplatin or carboplatin for six cycles. ORR, PFS, and OS were 26%, 7.1, and 10.5 months, respectively. Authors reported mainly hematological toxicity, experiencing grade 3–4 neutropenia and thrombocytopenia in 52% and 43% of patients, respectively [56].

TKIs represent a valuable option for the treatment of metastatic ccRCC, however no results from CDC-specific prospective phase III trials are available so far. Retrospective data reported encouraging results on the activity of different TKIs including sunitinib, sorafenib, pazopanib, cabozantinib, and the mTOR inhibitor temsirolimus [32,57,58,59]. The combination of sorafenib and chemotherapy with cisplatin plus gemcitabine reported an ORR of 30.8% and a disease control rate (DCR) of 84.6% in previously untreated mCDC patients [60]. A phase II study of sunitinib in patients with metastatic ncRCC conducted by Tannir et al., included six patients with mCDC or medullary carcinoma [48]. No ORR were observed, whereas four patients achieved disease stabilization and two patients experienced disease progression [48]. The only prospective trial evaluating efficacy and safety of the targeted agent cabozantinib as first line treatment for mCDC is the BONSAI trial. This Italian monocentric phase II trial enrolled 23 untreated mCDC patients. The study design was based on a Simon’s two stage optimal design. In detail, at least 2 responses in 9 pts in the first stage were needed to proceed to the second stage where at least 6 responses in 14 additional pts were needed to prove activity of cabozantinib. The study met its primary endpoint showing promising efficacy and acceptable tolerability of cabozantinib in mCDC pts. The authors will present mature results according to mutational profiles and gene signatures [61].

Due to the rarity of CDC, case reports furnish useful experiences. Bronchud et al. described an interesting case of clinical and radiological responses in a mCDC patient with high disease burden at diagnosis with HER2 overexpression on the primary tumor with the oral capecitabine together with double HER2 blockade with both intravenous trastuzumab and oral lapatinib [62].

To our knowledge, there are no immunotherapy trials specifically designed for CDC. Different case reports described the safety and activity of the immune-checkpoint inhibitor in previously treated mCDC patients. Among others, responses to nivolumab, a monoclonal antibody directed against the programmed cell death-1 (PD-1) or atezolizumab, a monoclonal antibody directed against programmed cell death ligand-1 (PD-L1) were reported [63,64,65]. A phase IIIb study of atezolizumab enrolling 1004 patients with locally advanced or metastatic pre-treated urothelial or non-urothelial carcinoma of the urinary tract, included eight CDC patients. Unfortunately, data regarding the outcome of CDC patients are not available. Overall, 8% of patients discontinued because of toxicity and 13% of Grade ≥3 adverse events were treatment-related, median OS was 8.7 months (95% CI 7.8–9.9), median PFS of 2.2 months (95% CI 2.1–2.4), and ORR of 13% (95% CI 11–16%) [66]. Immunotherapy combinations such as nivolumab in combination with cabozantinib or nivolumab plus ipilimumab in combination with cabozantinib are under evaluation. Completed and ongoing clinical trials evaluating treatments for mCDC patients are summarized in Table 3 and Table 4.

### 3.4. Sarcomatoid

No standard of care exists for sarcomatoid RCC. The International Metastatic Renal Cell Carcinoma Database Consortium (IMDC) collected data of 2286 patients, of whom 230 had sarcomatoid features. More than 93% of all patients received TKIs, experiencing a PFS of 4.5 months and an OS of 10.4 months [67].

Since sarcomatoid tumors express a high level of PD-1 and PD-L1, immunotherapy represents a promising therapy for these patients. The KEYNOTE 426 trial, testing axitinib plus pembrolizumab versus sunitinib, included 105 patients with sarcomatoid dedifferentiation. In this trial, the combination of axitinib plus pembrolizumab showed improved OS (HR 0.58, 95% CI 0.21–1.59; 12-month rate 83.4% vs. 79.5%), PFS (HR 0.54, 95% CI 0.29–1.00; median not reached vs. 8.4 mo), and ORR (58.8% [95% CI 44.2–72.4] vs. 31.5% [19.5–45.6]) in patients whose tumors had sarcomatoid features [68]. A post hoc analysis of CheckMate 214 was conducted focusing on intermediate-poor risk, advanced clear-cell RCC patients with sarcomatoid features. The descriptive analyses performed at a minimum follow-up of 30 months, confirmed promising efficacy in terms of ORR (56.7% versus 19%) and complete response rate (18.3% versus 0), OS (31.2 versus 13.6, HR 0.55), and PFS (8.4 versus 4.9 months, HR = 0.61) with nivolumab plus ipilimumab compared to suninitib in previously untreated, intermediate-poor risk, advanced clear-cell RCC with sarcomatoid features. In the primary analysis, treatment-related adverse events (TRAEs) occurred in 93% of patients treated with nivolumab plus ipilimumab and in 97% of patients treated with sunitinib. Grade 3 or 4 adverse events occurred in 46% and 63% of patients, respectively. TRAEs leading to discontinuation occurred in 22% and 12% of the patients in the respective groups. No new safety alarms emerged during the long-term follow-up [37]. Concerning the issue of survival in patients who had to discontinue immunotherapy for TRAEs, Tannir et al. conducted a post hoc analysis, showing that a OS benefit persisted in patients despite therapy discontinuation due to adverse events [38].

Recently, a phase II study provided the basis for considering bevacizumab and erlotinib as a valuable alternative in patients with HLRCC or sporadic papillary renal cell cancer, a population that has no widely accepted standard therapeutic options. A total of 83 patients were enrolled, including 42 in the HLRCC cohort and 41 in the sporadic cohort. The median PFS was 14.2 months (95% CI, 11.4–18.6) in all patients, 21.1 months (95% CI, 15.6–26.6) in the HLRCC cohort, and 8.7 months (95% CI, 6.4–12.6) in the sporadic cohort. The majority of registered TRAEs were grade 1 or 2, with the most common being acneiform rash (92%), diarrhea (77%), proteinuria (71%), and dry skin (61%). Grade ≥ 3 TRAEs occurred in 47% of patients, including hypertension (34%) and proteinuria (13%), with only one patient (1.2%) experiencing a grade 5 GI hemorrhage possibly related to bevacizumab [69].

### 3.5. Other Histological Types

We have no approved therapy specifically indicated for patients with Xp11 translocation mRCC. The major clinical efficacy trials have not established the percentage of patients with Xp11 translocation mRCC and thus it is difficult to establish drug efficacy in patients with this tumor subtype [39]. The first evidence of clinical activity of targeted therapy in patients with Xp11 translocation mRCC, is a case report of a male of 23-years old which found no clinical evidence of activity. Malouf et al. selected from the kidney tumor registries of the Juvenile RCC Network, 21 patients with Xp11 translocation/TFE3 fusion gene metastatic RCC who had received targeted therapy. The patients included displayed aggressive disease with a median PFS of 2 months when receiving a cytokine-based regimen and an 11% response rate. On the other hand, targeted therapy achieved objective responses and prolonged PFS In Xp11 translocation RCC similar to those reported for clear-cell RCC [39]. Moreover, TFE3 fusions characterizing Xp11 translocation RCC, result in an activation of MET signaling by transcriptional up-regulation that make these tumors probably responsive to therapeutic MET Inhibition [70].

Patients with HLRCC presented with reduced or absent activity of fumarate hydratase, with consequent fumarate-accumulation that generates a pseudo-hypoxic state with HIF-1 up-regulation. HIF-1 has been increasingly studied because of its perceived therapeutic potential [40]. These insights could further rationalize the use of personalized therapies or according to the individual genomic alterations.

RMC is another rare and aggressive form of kidney cancer. Treatment options are limited, as most standard therapies have not been found to be efficacious in RMC. Neither systemic therapy nor radiation therapy has been found to be particularly efficacious in the treatment of RMC. Despite the lack of available prospective evidence and the modest short-term palliation, targeted therapies and cytotoxic chemotherapy are the mainstay of treatment of RMC. A variety of chemotherapies has been tried such as cyclophosphamide, doxorubicin, cisplatin, topotecan, methotrexate, and vinblastine. Currently, no regimen has significantly improved outcomes [71]. In a phase 2 trial of bortezomib (a drug approved for the treatment of multiple myeloma and mantle cell lymphoma that targets the 26S proteasome of the ubiquitin-proteasome degradation system) in mRCC, one patient with mRMC achieved a complete remission and remained without evidence of disease after more than 27 months of follow-up [72]. Hopefully, improved understanding of the underlying biologic mechanisms of this rare disease will provide guidance for future therapies.

## 4. Discussion

ncRCC is a heterogeneous group of diseases including a number of histological subtypes that are disparate in presentation, clinical course, and genetic basis. Survival of all subtypes of ncRCC in the metastatic setting is uniformly worse than the clear-cell counterpart, and this can be explained considering the aggressiveness of these diseases, and lack of effective systemic treatment options [2]. Currently, there is no globally accepted standard of care for ncRCC. Therefore, enrolment into specific clinical trials is strongly recommended. Due to its rarity and aggressiveness, ncRCC has been often excluded from randomized phase II-III trials. Therefore, treatment choices are based on extrapolating results from ccRCC trials, retrospective data, and subgroup analysis or case reports. Most of the available data look at patients with papillary and chromophobe tumors. Data on other histologies are very limited. Based on all trials mentioned above, everolimus, sunitinib, cabozantinib, and bevacizumab are included in the treatment recommendations for ncRCC [73]. Overall, the most robust data exist on the use of sunitinb. Moreover, encouraging clinical results on checkpoint inhibitor activity have been recently reported supporting their use in ncRCC [53,54,63,64,65]. In addition to these general recommendations, some specific situations should be considered. Activating mutations or amplifications in *MET* are common in patients with PRCC type 1, more than type 2. Thus, cMET inhibitors such as cabozantinib appear as an acceptable option instead of the usual VEGF TKIs for mPRCC patients [22,23,24,25,26,27,28]. Further studies combining MET inhibitors with immunotherapeutic agents are ongoing to provide better outcomes for these patients. Moreover, type 2 tumors were characterized by CDKN2A silencing, SETD2 mutations, TFE3 fusions and increased expression of the NRF2-antioxidant response element (ARE) pathway. All these alterations represent potential targets of future personalized therapies [13]. In particular, *CDKN2A* alteration could confer sensitivity to CDK4/6 inhibitors (e.g., palbociclib, ribocivlib, or abemaciclib) [13]. Patients with chRCC RCC may benefit from mTOR inhibitors due to the mutation on chromosome 7 commonly described in these tumors, determining a loss of folliculin gene with upregulation of mTOR [31]. Sarcomatoid tumors are very inflamed tumors and sensitive to immune checkpoint inhibitors. Thus, the use of nivolumab/ipilimumab combination should be considered as a good option for these patients [37,38]. Finally, CDC patients may be considered for chemotherapy due to the similarities with urothelial carcinoma. However, evidence was reported on mutation in *NF2*, *SETD2*, *SMARCB1,* and *MLL* gene, as for ccRCC. The use of targeted therapies could be therefore considered as a good option for these patients [33,34]. Moreover, several trials are ongoing to test immunotherapy in ncCDC to help guide more targeted treatments (Table 3).

The gap in the clinical management of ncRCC is certainly due to the poor molecular characterization that prevents the development of tailored treatments for these tumors. Only a few studies have examined the genomic and transcriptomic landscape of ncRCC. In this review, we summarized pathogenetic mechanisms of ncRCC and the evolution of treatment paradigms over the last few decades, focusing on immune and targeted agents. However, evidence often reports heterogeneous results, that might be related to the broad molecular landscape of different subtypes. Genomic analysis highlighted that ncRCC are morphologically distinct from ccRCC and from each other, and their molecular characterization could reflect phenotypic differences, possibly resulting in a significant impact on clinical and therapeutic management and future clinical trial design. The molecular characterization of ncRCC subtypes should in fact lead to more appropriate clinical management and development of more effective forms of therapy. More efforts are certainly needed to identify novel molecular targets and to validate biomarkers suitable for the stratification of patients and the identification of better responders.

## 5. Conclusions

ncRCC is a heterogenous group of diseases made up of a number of different types of cancer classified by histology that are disparate in presentation, clinical course, and genetic basis. Due to its rarity and poor molecular characterization, ncRCC are underrepresented in clinical trials often managed with untailored treatments. More knowledge about the biology of these histologies is certainly needed. Histology-specific collaborative trials and controlled biomarker-based clinical trials are underway to establish a standard of care for these tumors.

## Figures and Tables

**Table 1:** Summary of key genetic and cytogenetic alterations in nc-RCC. PRCC: papillary renal cell carcinoma. CDC: collecting duct carcinoma. HLRCC: Hereditary leiomyomatosis and renal cell carcinoma.

Histological Type	Marker	Genetic Alteration	Clinical Significance
PRCC	3q,7,8,12,16,17,20 Y 8q 1p,9p	Trisomy Loss Gain Loss	
Type 1 PRCC	MET	Proto-oncogene germline alterations Somatic mutations	Hallmark of familiar forms Possible pharmacological target
Type 2 PRCC	CDKN2A SETD2 TFE3 NRF2	Silencing Somatic mutation Fusion Over-expression	
Chromophobe	7 TP53 PTEN	Somatic mutation	Possible target of mTOR inhibitors
CDC	NF2 SET2 SMARCB1 CDKN2A MLL SCL7A11	Somatic mutation Somatic mutation Somatic mutation Homozygous deletion Recurrent mutation Over-expression	Possible target of mTOR inhibitors Cisplatin-resistance marker
Sarcomatoid RCC	PDL-1	Over-expression	Increased susceptibility to ICIs
Xp11 translocation			
HLRCC	HIF-1	Up-regulation	Possible pharmacological target

**Table 2:** Completed prospective trials evaluating treatment regimens for papillary renal cell carcinoma patients. ORR: objective response rate; PFS: progression free survival; mo: months; OS: overall survival; Pap: papillary; Chr: chromophobe; Uncl: unclissified; Trasl: translocation; S: Sarcomatoid; NC: not calculated; CI: confidence interval; NR: not reached.

Trial	Treatment	Population (*n*)	ORR (%)	PFS (mo)	mOS (mo)
ASPEN	Sunitinib or Everolimus	Pap (65) Chr (20) Uncl (16) Trasl (12) S (11)	18 vs. 9	8.3 vs. 5.6 (95% CI 1.03–1.92)	32 (95% CI 15–NR) vs. 13 mo (95% CI: 10–38) HR 1.12 *p* = 0.60
SUPAP	Sunitinib	Pap 1 (15) Pap 2 (46)	13 (Pap 1) vs. (Pap 2)	6.6 [(95% CI 2.8–14.8) in Pap 1] vs. 5.5 [(95% CI 3.8–7.1) in Pap 2]	17.8 [(95% CI 5.7–26.1) in Pap1] vs. 12.4 [(95% CI 8.2–14.3) in Pap 2]
SAVOIR	Savolitinib or Sunitinib	Pap (180)	27 vs. 7	7.0 (95% CI, 2.8-NC]) vs. 5.6 (95% CI, 4.1–6.9)	NR (95% CI 11.9-NC) vs. 13.2 (95% CI, 7.6–NC)
PAPMET	Sunitinib or Cabozantinib or Savolitinib or Crizotinib	Pap (147)	23 (cabozantinib) vs. 4 (sunitinib)	9.0 [(95% CI 6–12) for cabozantinib] vs. 5.6 [(95% CI 3–7) sunitinib]	-
KEYNOTE 427 (COHORT B)	pembrolizumab	Pap (118) Chr (21) Uncl (26)	28 (Pap), 9.5 (Chr), 30.8 (Uncl)	-	-
CALYPSO	Savolitinib plus durvalumab	Pap 42	32	5.3 (95% CI 1.5–12.0)	NR

**Table 3:** Ongoing clinical trials evaluating treatment regimens for non-clear renal cell carcinoma patients. CDC: collecting duct carcinoma; ORR: objective response rate; Cc: clear cell renal carcinoma; cell; Pap: papillary renal cell carcinoma; Chr: chromophobe; Trasl: microphthalmia-associated transcription (MiT) family translocation renal cell carcinoma; Med: renal medullary carcinoma; uncl: unclassified carcinoma; S: renal cell carcinoma with a prominent sarcomatoid component; Rhab: renal cell carcinoma with a prominent rhabdoid component; FHD: Fumarate Hydratase Deficient Renal Cell Carcinoma; SDD: Succinate Dehydrogenase Deficient Renal Cell Carcinoma; SSE-FS: 1. Symptomatic skeletal event (SSE)-free survival (FS).

Trial	Phase	Treatment	Population	Line	Estimated Patients (*n*)	Primary Endpoint
NCT02363751	II	bevacizumab plus gemcitabine and platinum salts	CDC	I	41	ORR, 6-months PFS
NCT04071223	II	Radium Ra 223 dichloride plus Cabozantinib or Cabozantinib	Cc, Pap, Chr, Trasl, CDC, Med, Uncl, S, Rhab	I or further	210	SSE-FS
NCT03012581	II	Nivolumab	Pap, Uncl, Chr, Med, CDC, Uncl, S	II or further	300	ORR
NCT03635892	II	Cabozantinib plus Nivolumab	Uncl, Pap, FHD, SDD, CDC, Chr	I or further	57	ORR
NCT04413123	II	Cabozantinib plus Nivolumab and Ipilimumab	Uncl, Pap, Trasl, CDC, Med, Chr	I or further	40	ORR
NCT04267120	II	Pembrolizumab plus Lenvatinib	Pap, Chr, Trasl, SDD, S, Uncl	I	34	ORR

**Table 4:** Completed clinical trials evaluating treatment regimens for mCDC patients. ORR: objective response rate; PFS: progression free survival; DR: drug-related; AEs: adverse events.

Authors	Phase	Treatment	Line	Patients (*n*)	Primary Endpoint	Results
CHEMOTHERAPY						
Oudard et al. (2007) [56]	II	gemcitabine plus cisplatin or carboplatin	I	23	ORR	26%
Tannir et al. (2012) [48]	II	sunitinib	Further	57 *	ORR	0
Sheng et al. (2018) [60]	II	sorafenib plus gemcitabine and cisplatin	I	26	PFS	8.8 months
TARGETED AGENTS:						
Armstrong AJ (2016) [47]	II	Everolimus or sunitinib	II	108	PFS	8.3 vs. 5.6 months, Hazard ratio 1·41 [80% CI 1·03–1·92]; *p* = 0·16
IMMUNE-CHECKPOINT INHIBITORS:						
Sternberg et al. (2019) [66]	IIIb	atezolizumab	II or further	1004 ^§^ (8 CDC)	Safety	13% Grade ≥ 3 DR AEs
Procopio et al. (2021) [61]	II	cabozantinib	I	23	ORR	35%

* The trial enrolled all non-clear cell histologies: papillary, 27; chromophobe, 5; unclassified, 8; collecting duct or medullary carcinoma, 6; sarcomatoid, 7; and others, 4. ^§^ The trial allowed the inclusion of patients with urothelial or non-urothelial carcinoma of the urinary tract.
